# Clinicopathological Characteristics and the First Mutational Analysis of Gastrointestinal Stromal Tumors From Mexico: A Single Institution Experience

**DOI:** 10.7759/cureus.62594

**Published:** 2024-06-18

**Authors:** Rafael Medrano Guzman, Edgar F Perez Ventura, Atl Simon Arias Rivera, Patricia Piña-Sanchez, Moises Brener Chaoul

**Affiliations:** 1 Surgical Oncology, Unidad Médica de Alta Especialidad (UMAE) Hospital de Oncologia Centro Medico Nacional Siglo XXI, Instituto Mexicano del Seguro Social (IMSS), Mexico City, MEX; 2 General Surgery, Hospital Angeles Lomas, Huixquilucan, MEX; 3 Medical Research Unit in Oncological Diseases, Unidad Médica de Alta Especialidad (UMAE) Hospital de Oncologia Centro Medico Nacional Siglo XXI, Instituto Mexicano del Seguro Social (IMSS), Mexico City, MEX; 4 Surgical Oncology, Hospital Angeles Lomas, Huixquilucan, MEX

**Keywords:** platelet-derived growth factor receptor alpha (pdgfra) mutation, tumor, c-kit mutation, braf mutation, gastrointestinal stromal tumor (gist)

## Abstract

Background

Gastrointestinal stromal tumors (GISTs) arise from Cajal’s interstitial cell precursors and display a variety of genetic mutations, primarily in the *KIT* and *PDGFRA* genes. These mutations are linked to tumor location, prognosis, and response to treatment. This study delves into the mutational patterns of GISTs in a Mexican population and their impact on overall survival (OS) and disease-free survival (DFS).

Methodology

This retrospective study examined 42 GIST cases diagnosed at the Oncology Hospital of the National Medical Center XXI Century between January 2018 and December 2020. Clinical, histological, and immunohistochemical data were gathered, and mutational analysis of *KIT* and *PDGFRA* genes was conducted using second-generation sequencing.

Results

The study group consisted of 52.4% females and 47.6% males, with an average age of 62.6 years. The most common tumor site was the stomach (59.5%), followed by the small intestine (26.2%). *KIT* mutations were detected in 71.4% of cases, predominantly involving exon 11. *PDGFRA* mutations were observed in 7.1% of cases. Recurrence was noted in 9.5% of patients, all with high-risk tumors. No significant link was identified between specific mutations and OS or DFS.

Conclusions

This investigation sheds light on the genetic landscape of GISTs in the Mexican population. While no significant association was established between particular mutations and survival outcomes, the study emphasizes the importance of molecular profiling in treatment decision-making. Further studies with larger sample sizes and longer follow-up periods are necessary to validate these results and explore their clinical relevance.

## Introduction

Gastrointestinal stromal tumors (GISTs) are derived mainly from the precursors of interstitial cells of Cajal, which are responsible for intestinal motility. Although those with *PDGFRA*-type mutation can be derived from telocytes, tumors with *BRAF*-type mutation can be derived from smooth muscle cells [1,2]. It comprises a heterogeneous group of tumors with a molecular variety of mutually exclusive activated oncogenes, mainly *KIT* and *PDGFRA*. Incidence varies from 6 to 22 cases per 1,000,000 individuals per year depending on the region. Most patients present with gastrointestinal symptoms such as bleeding (>50%), obstruction (35%), and pain (20%). Moreover, 60-65% of GISTs are gastric, 20-25% are of the small intestine, and there are other less common locations such as the rectum (3-5%), colon (1-2%), and esophagus (1%). Patients are diagnosed at an average age of 65 years. In children, GISTs without *KIT* or *PDGFRA* mutations (wild-type GIST) are more common, as opposed to individuals over the age of 18 who are more likely to have mutations in *KIT* [3-13].

Few studies have documented the molecular incidence of GISTs. It is estimated that *KIT* mutations have an incidence of 8 cases per 1,000,000 individuals per year depending on the region, while the incidence of *PDGFRA* mutations is <3 cases for 1,000,000 people per year [9,14].

Usually, the type of mutation correlates with the anatomical site. Tumors with exon 9 *KIT* mutations occur in the small intestine, colon, or rectum. On the other hand, tumors with *PDGFRA* mutations, with the most common being D842V, occur in the stomach. GISTs with mutations in the succinate dehydrogenase complex (SDH) tend to be multifocal, although they commonly present as gastric tumors of young patients with predominance in women [15-17].

Most mutations occur in *KIT* (60-70%) or *PDGFRA* (10-15%). Approximately 15% of patients do not have *KIT* or *PDGFRA* mutations but have other genetic alterations such as mutations in the family of the genes of *SDH*, *RAS*, *BRAF*, *NF1*, or some even more rare as genetic fusion involving *NTRK3* or *FGFR1* [5,13,16,18-20].

Overall, 95% of GISTs are estimated to be positive for the CD117 protein (product of the gene *KIT* 4q12, type III tyrosine kinase receptor). The most common mutations in *KIT* are in exon 11 (66-71%), exon 9 (10-20%), and exon 13, 14, and 17 (1% each). The mutations found in exon 11 are mainly found between codons 550 and 579, in particular, in codons 557-559. Exon 9 mutations have been identified in residues 502-503, associated with high malignant potential [21].

These types of mutations are predictive factors of response to therapy, specifically to imatinib. Patients with exon 11 mutation had a better response, measured as overall survival (OS), compared to patients with mutations in exon 9 or wild phenotype [12,22].

Almost all GISTs can recur after complete surgical resection of the primary tumor. The five-year recurrence rate is more than 50%, with an average recurrence-free survival of 18 to 24 months. The most frequent sites of recurrence are the liver (67%), followed by the peritoneum. The five-year survival rate is 35-65%. In patients with irresectable disease, average survival is 10-20 months [23,24].

The importance of predictive factors lies in the ability to predict the risk of recurrence and the chances of survival more accurately. To date, the most effective treatment is surgical resection with negative margins in the localized primary disease. After a primary resection, the disease-free survival (DFS) at five years is 96% for low-risk GIST patients, 54% for intermediate-risk patients, and 20% for high-risk patients. The average time for recurrence is 19-25 months [25].

Conducting both *KIT* and *PDGFRA* tests allows us to plan treatment with tyrosine kinase inhibitors (TKIs), as these correlate with response or lack of response with specific TKIs. Different imatinib response rates have been correlated based on their mutational status. *KIT* mutations in exon 9 have lower response rates and SLP than those in exon 11 at 400 mg daily doses, whereas 400 mg dose every 12 hours has been associated with better SLP [26]. Most mutations in *PDGFRA* are associated with imatinib response, except for the mutation in D842V, which uncommonly responds to imatinib, but responds to other TKIs such as avapritinib.

Reports of experience in Mexico in the treatment of GISTs are scarce. To date, there are two series of cases, the first from the National Institute of Cancerology of Dr. López-Basave conducted in 2007. In total, 17 cases of GIST were identified through clinical records between 1995 and 2005, with the relative three-year survival rate for the entire cohort being 29.4% [27]. Another series of 62 patients from the National Institute of Nutrition “Salvador Zubirán” was reported by Dr. Medina-Franco in 2009. The small intestine was the most frequent localization, with an average follow-up of 37 months. The overall DFS at five years was 76% and 59%, respectively.

Unfortunately, research in Mexico on this subject is scarce, and there is no study describing mutations in Mexico. This study provides an opportunity to analyze this topic in a Mexican population to generate new information and address questions that have not yet been resolved. This study aimed to evaluate the mutational profile in patients with GISTs and their association with OS and DFS after undergoing primary surgical resection in our institution [23].

## Materials and methods

Studio design

This was an observational, longitudinal, retrospective, and analytical study. The cases were obtained through the Pathological Anatomy Service of the oncology hospital of the National Medical Center of the twenty-first century. The cases with primary GISTs that were immunohistochemistry positive for CD117, DOG1, and CD34 were identified retrospectively between January 2018 and December 2020. Information was collected from the clinical records of such cases where the variables of sex, age, type of resection, site of the primary tumor, histological type, mitotic index, histologic degree, necrosis, margins, ganglion status, clinical stage, immunohistochemical profile, and adjuvant treatment received by patients were recorded. The paraffin sheets and blocks from these cases were collected for the study of mutations by sequencing. The *KIT* and *PDGFRA* mutations were analyzed.

Procedure

DNA was extracted using the DNA FFPE Tissue (QIAmp) kit. Subsequently, the DNA obtained was quantified to evaluate the concentration and quality. First through spectral photometry in the EPOCH equipment, the samples needed to be more than 50 ng/µL in concentration, purity 269/280 1.9-2.0, and ratio 260/230 >1.0. Samples that passed the first quality filter were quantified again by fluorescence (QUBIT dsDNA HS), and samples with concentrations greater than 10 ng/µL were selected for library realization. Additionally, agarose gels were used to visualize DNA integrity.

Second-Generation Sequencing

It was carried out using AmpliSeq by ILUMINA Focus Panel to analyze mutations of interest in the KIT and PDGFRA genes. Second-generation mass sequencing was characterized by simultaneously carrying out multiple parallel sequence reactions. In general, it can be divided into the three steps described below.

Preparation of libraries: Amplification of white (Ampliseq Focus DNA Panel) by polymerase chain reaction (PCR) was performed. Then, partial digestion of amplicons (FuPa) was performed by the linking of the index adapters (Ampliseq CD Indexes y DNA ligase). The indices were previously mixed into a single-use board to ensure unique combinations. Subsequently, the bookstores were cleaned with magnetic pearls (AMPuere XP Beads).

Amplification of libraries: This was performed using PCR (Amp Mix and Library Amp Primers). The libraries were then cleaned using magnetic beads (AMPure XP Beads) and the quality was analyzed. The libraries with optimal quality of the fragments were assessed using a Bioanalyzer (Agilent 2100 Bioanalyzer). If fragments were between 197 and 277 pb, they were considered optimal and diluted to the required concentration for subsequent sequencing. For the Miseq system, a 2 nM concentration and a 1.1-1.9 pM final load concentration were used.

Sequencing through different technologies: The technology used in the MiSeq AmpliSeq by ILUMINA Focus Panel can detect mutations from a panel of genes of interest in solid tumors, including *KIT* and *PDGFRA*. The mutations that were identified were single-nucleotide variations, inserts/deletions, and variants of the number of copies.

Statistical analysis

The following methods of descriptive statistics were used: frequency tables, contingency tables, comparative bar charts of averages, and statistical summary measurements (average, median, standard deviation, and range). The statistical inference methods included parametric variance analysis, frequency data analysis (chi-square independence test), Kaplan-Meier survival analysis (log-rank test), and 95% confidence intervals (CIs) for the average. Actuarial global survival and DFS analyses were calculated for 12, 36, and 60 months. The SPSS (IBM Corp., Armonk, NY, USA) software was used for the analysis.

Ethical considerations

The present observational and retrospective study is considered risk-free research for the individuals subject to the study, as the information collected was based on records, and no intervention or modification of the individual variables was performed. Hence, it has no ethical implications sanctioned by the 1964 Helsinki World Medical Assembly or the 1949 International Code of Medical Ethics, as well as by the 1984 General Health Act or the 1987 Regulations of the General Health Law on Health Research.

For the analysis of retrospective cases, exemption from informed consent was requested because the identification of mutations was performed for descriptive purposes using material archived in the pathology files from patients who had already been diagnosed and treated, such that the determination of the mutations did not modify the treatment. It is worth mentioning that every five years all cases filed in pathology of the UMAE Hospital of Oncology are cleared, so with this work, information will be obtained on the biological material that in five years will be outdated. This ensures the anonymity of cases (they are encrypted with a consecutive internal record that does not allow the identification of sensitive patient information) and confidentiality of information. The study received the approval of the ethics committee of the unit.

## Results

Of the 42 patients included in the study (Table 1), 22 (52.4%) were females, and 20 (47.6%) were males. Age showed a normal distribution according to the Kolmogorov-Smirnov test (p = 0.2), with an average of 62.60 ± 11.6 years, a median of 63.5 years, an interquartile range of 56 to 70 years, a minimum of 28 years, and a maximum of 87 years.

**Table 1:** Clinical and pathological characteristics of patients.

Parameter		
Sex	Male	20 (47.6%)
	Female	22 (52.4%)
Age	Average	±11.6 (62.6%)
Primary tumor site	Gastric	25 (59.5%)
	Intestinal	11 (26.2%)
	Duodenal	4 (9.5%)
	Retroperitoneal	1 (2.4%)
	Right colon	1 (2.4%)
Adjuvant	Yes	28 (66.7%)
	No	14 (33.3%)
Tumor size	Average	±6.1 (10.3%)
Histological type	Fusocellular	23 (54.8%)
	Mesenchymal	10 (23.8%)
	Epithelioid and fusocellular	6 (14.3%)
	Epithelioid	2 (4.8%)
	Chondroid	1 (2.4%)
Histologic grade	G1	23 (54.8%)
	G2	1 (2.4%)
	G3	18 (42.9%)
Margins	R0	40 (95.2%)
	R1	1 (2.4%)
	R2	1 (2.4%)
Risk	Low	9 (21.4%)
	Moderate	8 (19%)
	High	25 (59.5%)

The site of the primary tumor was gastric in 25 (59.5%), intestinal in 11 (26.2%), and duodenal in four (9.5%) patients. The type of first resection performed in patients was the gastric wedge in 15 (35.7%) cases, followed by the intestinal resection in 10 (23.8%), and total gastrectomy in five (11.9%). The other procedures that had a frequency of fewer than five cases included subtotal gastrectomy, the Whipple procedure, gastric wedge plus splenectomy, hemicolectomy, and multi-structural and retroperitoneal resection. Subsequently, the tumor size was evaluated, which showed an average of 10.3 ± 6.1 cm, a median of 9 cm, an interquartile range of 6.8 cm to 12.6 cm, a minimum of 3.8 cm, and a maximum of 37 cm (normality test, p = 0.02).

The adjuvance which was described as post-primary treatment was performed in 28 (66.7%) cases. Imatinib 400 mg was administered in 26 (61.9%) patients, imatinib 800 mg in one (2.4%) patient, and sunitinib 25 mg in one (2.4%) patient. Neoadyuvancy was performed in two (4.8%) patients, with both receiving imatinib 400 mg over 6 and 10 months, respectively. According to the histological type in each participant, the fusocellular type was seen in 23 (54.8%) patients, mesenchymal in 10 (23.8%), and epithelial and fusocelular in six (14.3%). The histological grade obtained the following distribution: grade 1 in 23 (54.8%), grade 2 in one (2.4%), and grade 3 in 18 (42.9%). According to resection margins, R0 was seen in 40 (95.2%) patients and R1 and R2 in one (2.4%) patient each. Risk assessments were divided into high, moderate, and low accounting for 25 (59.5%), eight (19%), and nine (21.4%) cases, respectively.

Of a total of 33 participants, 78.6% did not have nodules. In the remaining nine cases, the nodules evaluated were two, three, four, six, seven, nine, 18, 22, and four, corresponding to one (2.4%) each. Of them, only one participant had positive nodules. Metastases were evaluated in 11 (26.2%) cases, with the most common being liver metastases in six (14.3%), followed by sarcomatosis in two (4.8%), and pulmonary, liver, pancreatic, and peritoneal in one (2.4%) case each. For the immunohistochemistry profile, 42 (100%) participants were positive for CD117 and DOG, four (9.5%) for AML, and one (2.4%) for S100 (Table 2).

**Table 2:** Description of the immunohistochemistry profile.

Immunohistochemical profile	Positive	Negative
CD117	42 (100%)	0 (0%)
DOG1	42 (100%)	0 (0%)
CD34	10 (23.8%)	32 (76.2%)
AML	4 (9.5%)	38 (90.5%)
S100	1 (2.4%)	41 (97.6%)

**Figure 1:**
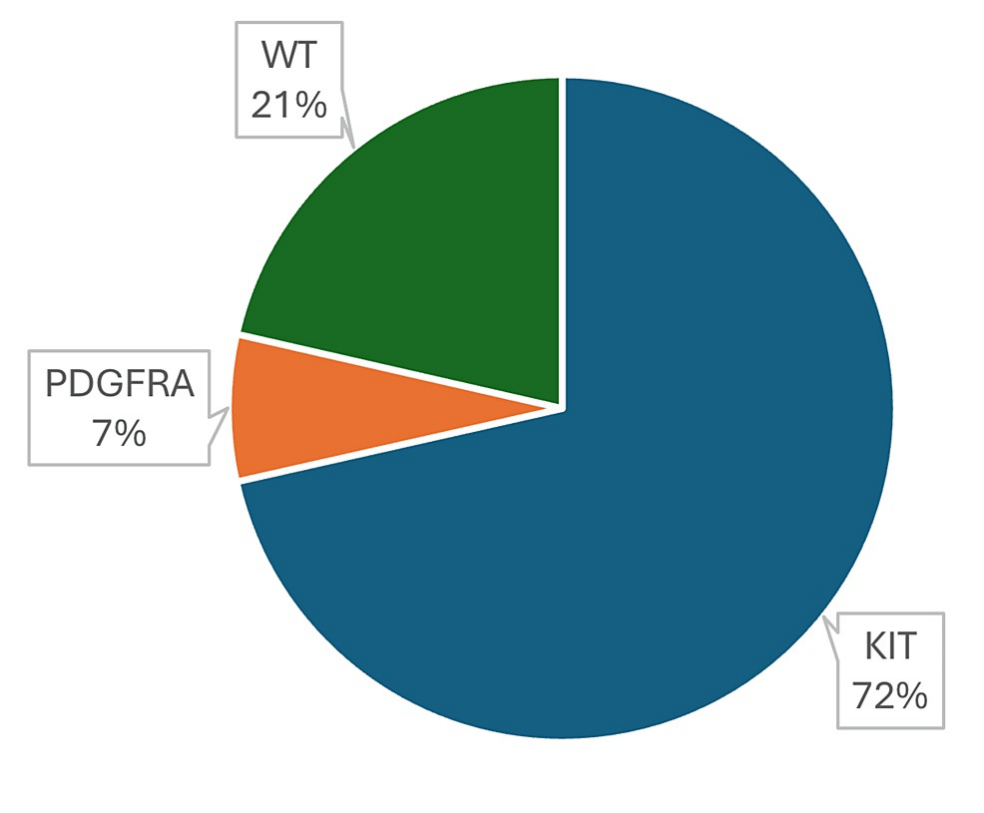
Percentage per mutated gene.

Information on the mutated gene was also obtained: 30 (71.4%) cases showed *KIT* mutations, nine (21.4%) were wild type, and three (7.1%) showed *PDFGRA* mutations (Figure 1).

The exons in which the mutation was found are detailed in Table 3.

**Table 3:** Description by mutated exons.

Exon	Frequency (n)	Percentage (%)
10	2	4.8
11	24	57.1
14	1	2.4
17	1	2.4
18	2	4.8
9	2	4.8
9, 10	1	2.4
WT	9	21.4

**Figure 2:**
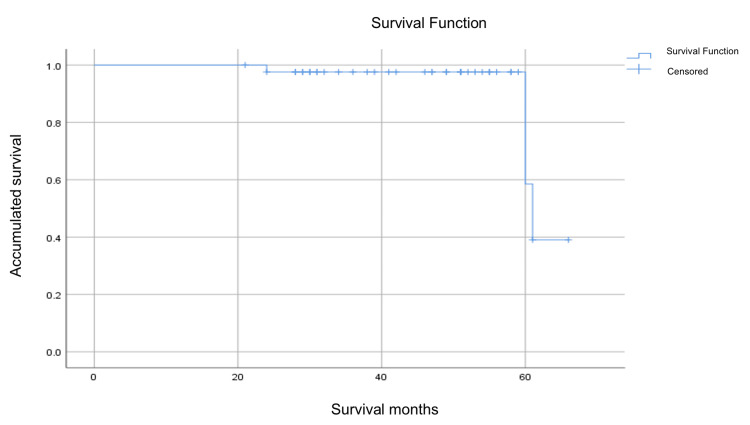
Kaplan-Meier survival for patients with gastrointestinal stromal tumor recurrence.

Recurrence was seen in four (9.5%) patients, of whom two (50%) were males and two (50%) were females. Survival was evaluated in each of the participants in which the follow-up time to the date of the last appointment obtained in the hospital was detailed, an estimate of 61 months was obtained with a 95% CI of 58.6 to 64.6 months. No deaths were recorded in the study population. The follow-up was continued in participants until the recurrence of the disease where only four patients presented two mutations in exon 11 and one in exon 9 (Figure 2).

**Figure 3:**
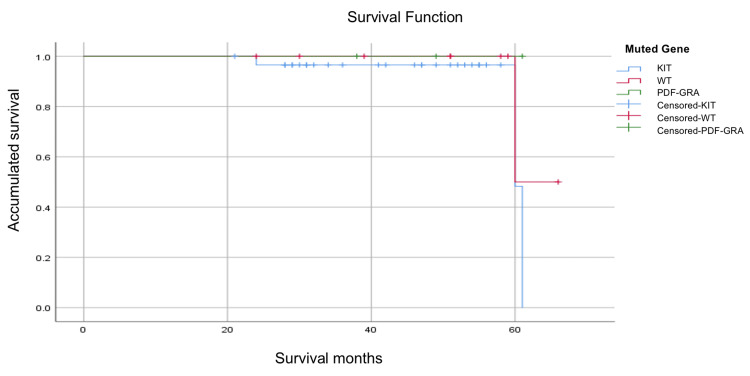
Kaplan-Meier survival for patients with recurrence according to the mutated gene.

Finally, it was assessed whether there was a difference between the mutated gene and disease recurrence. No statistically significant differences were obtained (p = 0.08). Patients with mutations were not included in *PDFGRA* (three patients) because they did not have recurrences (Figure 3).

## Discussion

GISTs are the most common mesenchymal tumors of the intestine, generally accounting for approximately 1% of all types of tumors. Several studies have reported an incidence ranging between 11 and 15 cases in 1 million patients; however, this depends on the population evaluated. Due to the presentation, it is often difficult to analyze large-scale populations because in many cases the diagnosis is incidental.

The number of cases currently has developed more research regarding this pathology. It has emerged that these tumors can occur anywhere in the digestive tract, and, according to some reports, the most prevalent tumor is in the stomach. However, it is important to obtain information on the tumor location due to the management, prognosis, and follow-up of the patients.

A study conducted in 2018 evaluated a total of 52 GIST cases from 2011 to 2016, where the characteristics of the tumors and the prognosis were recorded. The average age was 53.4 years, 61.5% were men, and metastasis developed in 7.7% of the population. The most common site was the stomach at 57.7%, followed by the small intestine at 19.2%. The tumor averaged 9.4 cm; however, 42.3% were greater than 10 cm. The most common histological type was fusiform at 75%. Overall, 55.8% were of high risk according to mitotic activity and 44.2% were of low risk. Necrosis was at 19.2%. Overall, 34 of 46 tumors were positive for CD34, 46 of 48 for CD117, and 12 of 40 were positive for S100 [28].

In comparison with our results, the average age was 62.6 years, where 75% of the population was between 56 and 70 years old, and the female sex was more frequent (52.4%). The site of the tumor was similar to the results obtained in the previous study with a total of 59.5%. The tumor size achieved an average of 10.3 cm with a median of 9 cm. The most common histological type was fusocellular with a total of 54.8%. Necrosis developed most frequently in our population with an overall rate of 47.6%. According to the immunohistochemical profile, CD34 was positive in 10 (23.8%), CD117 in 42 (100%), DOG4 in two (100%), AML in four (9.5%), and S100 in one (2.4%).

Joensuu investigated the effect of *KIT* and *PDFGRA* mutations on survival in patients with GIST treated with surgery and imatinib. Patients were randomized to receive imatinib for one or three years. During a median follow-up of 88 months, 142 patients had a recurrence of GIST. Compared with our results, over a follow-up of 61 months, four patients developed recurrence, 80.4% of patients had a *KIT* mutation, and 7% of patients had *PDFGRA* mutations. In general, the mutation in exon 11 was associated with a favorable recurrence-free survival and in exon 9 unfavorable. In our study, due to the sample size, reliable data cannot be obtained regarding the association of exon mutation and disease-free period; however, it is important to mention that of the total of patients who developed recurrence, four cases were of high risk according to mitotic index, all developed metastases, 100% were positive for CD117 and DOG1 and negative for CD34, three cases had *KIT* mutations, where two were in exon 11 and one in exon 9, with position 576, 554, and 502, respectively [22,29].

In general, as GISTs are uncommon, the timely detection, as well as the identification of histological and immunohistochemical characteristics, is fundamental for the follow-up of patients in the medium and long term to improve the disease-free period of patients and decrease mortality. In our study, there were no deaths due to this pathology. It is necessary to conduct more studies with a larger sample size and long-term monitoring to obtain reliable and representative results of the Mexican population.

The main limitation of this study was the size of our study sample. A larger patient sample should be included in a future study.

## Conclusions

No significant difference was noted between the association of mutations in GIST and OS and DFS. This study is the first from Mexico. These findings could have significant implications for the treatment and prognosis of patients with GIST. Hence, a larger study sample and follow-up time are required for an adequate evaluation of the mutational profile association and the above-mentioned factors.
